# Knowledge, Attitudes, and Behaviors of Parents towards Recommended Adult Vaccinations: An Explanatory Survey in the Geographic Area of Naples, Italy

**DOI:** 10.3390/ijerph16122070

**Published:** 2019-06-12

**Authors:** Francesco Napolitano, Giorgia Della Polla, Italo Francesco Angelillo

**Affiliations:** Department of Experimental Medicine, University of Campania “Luigi Vanvitelli”, Via L. Armanni, 5 80138 Naples, Italy; francesco.napolitano2@unicampania.it (F.N.); giorgia.dellapolla@unicampania.it (G.D.P.)

**Keywords:** behavior, knowledge, parents, recommended vaccinations, survey, vaccinations coverage

## Abstract

The purposes of this study were to explore the knowledge, attitudes, and behaviors towards the recommended vaccinations for adults between 19–64 years of age and the associated factors among parents. The survey was conducted from October to December 2018 among a sample of parents randomly selected from five preschools and primary, secondary, and high schools in the geographic area of Naples, Italy. The mean age of participants was 45.2 years (range 19–71). Only 16% of the parents knew all vaccinations recommended to adults between 19–64 years of age. Those being healthcare professionals, having a chronic condition, having received information about vaccinations from physicians, and having a lower educational level were more likely to know the vaccinations recommended to adults between 19–64 years of age. Female participants, those who had received information about vaccinations from physicians, and those who had a lower number of children were more likely to have a positive attitude toward the usefulness of the administration of vaccinations recommended to adults between 19–64 years of age. Among unvaccinated respondents, more than half reported a positive attitude toward willingness to receive a recommended vaccination. This positive attitude was significantly higher among those who considered vaccinations as being useful and among who had received information from physicians. Only 16.9% self-reported to have received at least one vaccination recommended to adults between 19–64 years of age. Those who were healthcare professionals, who had at least one chronic condition, and who considered the administration of the vaccinations as being useful were more likely to have received at least one recommended vaccination. Greater efforts by policy makers and healthcare providers are needed to increase parents’ knowledge on recommended vaccines, and it is also crucial that healthcare providers have a high knowledge and favorable attitudes in order to increase vaccine coverage.

## 1. Introduction

Several vaccination programs have been implemented all over the world for the individuals who due to epidemiological, health, occupational, or behavioral conditions are at higher risk of contracting infectious diseases [1]. Among these groups, it is well known that adults, especially those older or with chronic health conditions, are at higher risk of severe illness or complications from infection with rates of vaccination coverage also considerably lower than those established by the objectives of the immunization programs [2,3,4,5].

In Italy, specific non-obligatory vaccinations have been recommended to adults between 19–64 years of age. Indeed, they should receive every 10 years the combined vaccine against diphtheria, tetanus and pertussis; measles, rubella and mumps; varicella, and, for those of any age with chronic conditions, influenza with plans to extend this recommendation to those over 50 years of age [6]. Despite these recommendations, Italy has yet to implement effective policies. Indeed, reports indicate very poor vaccination uptake for influenza among those over 64 years of age (57.2%) [7]. Even if there are no doubts about the safety and efficacy of the vaccines, the decision to vaccinate is influenced by several determinants, including vaccine hesitancy, cost, access and availability of vaccines, and cultural beliefs [8,9,10,11,12]. In particular, vaccine hesitancy and the reluctance or refusal to vaccinate have been widely studied among different groups [8,13,14,15,16]. Moreover, Internet and social media have contributed to the production of mistrust [17,18,19,20] and, therefore, healthcare providers have a key role in order to improve vaccination coverage also among adults and a lack of knowledge acquisition is indicative of a gap in communication. Consequently, investigating the level of knowledge and attitudes of the adult population regarding the recommended vaccinations is crucial in order to develop effective strategies to improve the coverage. To the best of our knowledge, few studies have evaluated the level of knowledge and the acceptability of vaccinations among the adult population in Italy [21,22]. Understanding adults’ vaccination-related knowledge, attitudes, and behavior may provide useful information in order to implement targeted educational interventions and policies aimed at appropriate behaviors. Therefore, the purposes of the present cross-sectional study conducted among a large sample of parents in Italy were: (a) to explore the knowledge, attitudes, and behaviors towards the recommended vaccinations to adults between 19–64 years of age; and (b) to provide an insight into the factors influencing the knowledge, attitudes, and behaviors.

## 2. Materials and Methods

### 2.1. Study Population and Sampling

The survey was conducted from October to December 2018 in the geographic metropolitan area of Naples, Italy. This area has a territorial extension of 1171 km^2^, a population density of 2634/km^2^, an unemployment rate of 30.5%, and a lower income per capita (€ 12,755) compared to the national average. The participants have been recruited in this area because it includes more than three million people with similar socio-demographic characteristics compared to the whole population of the Campania Region. The sample consisted of 550 parents of children within the age group ranging from 3 to 18 years attending the selected schools and was selected through a two stage cluster sampling method. From the list of public preschools, primary, secondary, and high schools, five of them were randomly selected, and in the second stage approximately 20% of all children were selected through a simple random sampling from each school. The effective sample size was estimated to be 430 parents assuming a prevalence of 70% of parents who would have been vaccinated with the recommended vaccines, a 95% confidence interval, an error of 5%, and assuming a response rate of 75%. 

### 2.2. Data Collection

Prior to the start of the investigation, the research team contacted the heads of the selected schools by letter requesting permission to perform the study, clarifying the objectives of the investigation, the study methodology, and assuring the anonymity and confidentiality of the collected data. After approval was granted, the teachers gave a sealed envelope directly to the parents of the children in the preschools or to the children in the primary, secondary, and high schools. The envelope contained a research information sheet that described the study goals, contact details, and instructions to return the completed questionnaire, a two-page anonymous questionnaire to be completed by only one parent, an informed consent form, and two self-addressed envelopes for returning the questionnaire and the signed informed consent to the research team. Participants were also informed that all the data collected were processed and analyzed anonymously, that their responses were not linked with the participants’ identification, and that participation would be voluntary. The respondents did not receive any financial or other compensation for participating in the survey.

### 2.3. Survey Instrument

A standardized self-administered questionnaire divided into five parts was used. The first part asked about the participants’ socio-demographic characteristics and their health status (age, nationality, marital status, educational level, employment status, number of children, type and number of chronic conditions, self-reported health status). The self-reported health status was measured with a 10-point Likert scale ranging from 1 (bad) to 10 (excellent). In the knowledge section, respondents were asked a series of questions regarding the recommended vaccinations for adults between 19–64 years of age in Italy (vaccines available, number of doses and booster shot for each vaccination). Response options included “yes,” “no”, or “do not know” and free-text responses. The third part examined the parents’ attitudes about the vaccine-preventable infectious diseases and the vaccinations recommended to adults between 19–64 years of age (concern about the dangerousness of the diseases and of the recommended vaccines, usefulness of the vaccinations, willingness to receive or not receive vaccinations). The attitudes were measured with a 10-point Likert scale ranging from 1 to 10 with higher values corresponding to a stronger attitude. The fourth part asked about participants’ behaviors regarding vaccinations recommended to adults between 19–64 years of age (recommended vaccinations received, advice from physicians with regards to vaccines, reasons for having or not having received the vaccinations). Response options included free-text responses and selection from a list of choices. The fifth part asked whether they received information about vaccinations for adulthood and the sources of knowledge, and whether they needed to receive additional information. Response options included “yes” or “no” responses and selection from a list of choices. The questionnaire was pilot tested on 20 parents who were not included in the actual study.

The study protocol and the questionnaire were approved by the Ethics Committee of the Teaching Hospital of the University of Campania “Luigi Vanvitelli” (protocol number 634). 

### 2.4. Statistical Analysis 

All statistical analyses were performed with the software Stata version 15 [23]. Firstly, descriptive analysis has been performed in order to describe the principal characteristics of the sample. Secondly, chi-square and Student’s *t*-test were conducted for bivariate analysis to assess the association between each independent variable and the different outcomes of interest. All independent variables with a *p*-value equal or less than 0.25 at the bivariate analyses were included in the multivariate stepwise logistic and linear regression models. Multivariate stepwise logistic and linear regression models were constructed to identify factors associated with the following dichotomous and continuous outcomes of interest: knowledge of the vaccinations recommended to adults between 19–64 years of age (tetanus/diphtheria/pertussis, measles/mumps/rubella, varicella, and influenza for those with chronic conditions) (no = 0; yes = 1) (Model 1), having a positive attitude towards the usefulness of the administration of vaccinations recommended to adults between 19–64 years of age (continuous) (Model 2), having received at least one vaccination recommended to adults between 19–64 years of age (no = 0; yes = 1) (Model 3), and positive attitude toward willingness to receive vaccinations recommended to adults between 19–64 years of age (no = 0; yes = 1) (Model 4). The following independent variables were included in all Models: Age (continuous, in years), gender (male = 0; female = 1), educational level (college degree or higher = 0; none or primary/middle/high schools = 1), working activity as healthcare professional (no = 0; yes = 1), having at least one chronic condition (no = 0; yes = 1), self-rated health status (continuous), and having received information from physicians about the recommended vaccinations during adulthood (no = 0; yes = 1). Moreover, the variables number of children (continuous) and knowledge of the vaccinations recommended during adulthood (no = 0; yes = 1) were also included in Models 2 to 4; the variables considering the administration of the vaccinations as being useful (continuous), considering the administration of the vaccinations as being dangerous (continuous), and considering the vaccine-preventable infectious diseases during adulthood as being dangerous (continuous) in Models 3 and 4. 

The significant level choice for the inclusion and elimination of the variables in the multivariate stepwise logistic and linear regression models were respectively *p*-values of 0.2 and 0.4. Odds ratios (ORs) and 95% confidence intervals (CIs) were calculated in the logistic regression analysis. Standardized regression coefficients (β) were presented for the linear regression model. All statistical tests were two-tailed, and the level of statistical significance was set at *p*-value ≤ 0.05.

## 3. Results

A total of 412 parents responded to the survey for a response rate of 74.9%. The main socio-demographic characteristics of the sample are described in Table 1. A large majority was female, the average age was 45.2 years, almost all were married, the average number of children was 2, approximately one third had a college degree, and two-thirds were employed.

### 3.1. Knowledge

Of the 412 participants, a large majority (88.4%) had received information about vaccination from a variety of sources, including physicians (79.5%), mass media (18.9%), and the internet (11.6%). Moreover, more than half (53.4%) reported that they needed more education on the vaccinations recommended during adulthood.

Almost all correctly acknowledged the fact that vaccines against meningitis (99.7%), measles (98.3%), tetanus (95.9%), pertussis (92.5%), varicella (91.1%), influenza (86.6%), and hepatitis B (86.9%) are available in Italy. Moreover, a large majority were aware regarding the existence of the vaccines against human papillomavirus (66.3%), hepatitis A (63.6%), and tuberculosis (51.5%), and only one quarter (25%) indicated the vaccine against herpes zoster. Only a small proportion incorrectly answered that vaccines against hepatitis C (34.9%) and HIV (8.2%) are available. 

The results regarding the knowledge towards vaccinations recommended to adults between 19–64 years of age in Italy are presented in Table 2. Approximately one third knew that the vaccination against tetanus/diphtheria/pertussis (30.6%) was recommended for adults, whereas 26.7% and 19.7% correctly indicated measles/mumps/rubella and varicella, respectively. Moreover, more than one third correctly indicated that vaccination against influenza (35.4%) was recommended for adults with chronic conditions. In total, only 16% identified all vaccinations recommended during adulthood. 

Multivariate logistic and linear regression analyses were used to test whether key variables significantly predicted higher vaccination knowledge, positive attitudes, and high coverage rates and the results are showed in Table 3. Four variables in the model were significant predictors of knowledge: Educational level, health status, working activity, and source of information. Those being healthcare professionals (OR = 3.17; 95% CI 1.06–9.52), having at least one chronic condition (OR = 28.8; 95% CI 9.62–86.63), and having received information about vaccinations from physicians (OR = 2.68; 95% CI 1.01–7.09) were more likely to know the vaccinations recommended to adults between 19–64 years of age. Finally, respondents with a college degree were also significantly more likely to have a lower knowledge compared with those with a lower level of education (OR = 3.97; 95% CI 1.62–9.75) (Model 1).

### 3.2. Attitudes

With regard to attitudes, participants felt that the vaccine-preventable infectious diseases were dangerous for them (81%) with a mean value of 7.4 and the majority considered the vaccinations administered during adulthood as not being dangerous (58%) with an average value of 4.9. More than two thirds considered the administration of recommended vaccinations for adulthood as useful with a mean value of 7.4. The multivariate linear regression analysis showed that female participants, those who had received information about vaccinations from physicians, and those who had a lower number of children were more likely to have a positive attitude toward the usefulness of the administration of vaccinations recommended to adults between 19–64 years of age (Model 2 in Table 3). 

Among unvaccinated respondents, more than half (52.1%) reported a positive attitude toward willingness to receive a recommended vaccination. Multivariate logistic regression analysis showed that this positive attitude was significantly higher among those who considered vaccinations as useful (OR = 1.31; 95% CI 1.05–1.61) and among those who had received information about vaccinations from physicians (OR = 3.15; 95% CI 1.63–6.08) (Model 3 in Table 3). The most frequently reported reasons for the positive attitude toward willingness to receive a recommended vaccination were the usefulness of the vaccinations (76.6%), that the infectious diseases could be harmful for them (29.6%), and the recommendation from physicians (27.6%). Reasons for unwillingness to receive the vaccinations during adulthood included having an objection to the administration of the vaccines (77.9%), perceiving the vaccine-preventable infectious diseases as being non-dangerous (31.6%), and concerns about side effects of vaccines (9.1%). 

### 3.3. Behaviors

Only 16.9% of the participants self-reported to have received at least one vaccination recommended to adults between 19–64 years of age. The highest rates of coverage were for tetanus within the previous 10 years (41.6%) and influenza (40.7%) among those older than 50 years or with at least a chronic disease, whereas only 7.2% had been immunized during adulthood against measles/mumps/rubella and no one had been vaccinated against varicella. Those who were healthcare professionals (OR = 2.77; 95% CI 1.05–7.26), those who had at least one chronic condition (OR = 7.38; 95% CI 2.73–19.94), and those who considered the administration of the vaccinations useful (OR = 1.33; 95% CI 1.06–1.67) were more likely to have received at least one vaccination recommended to adults between 19–64 years of age (Model 4 in Table 3).

## 4. Discussion

This study investigated in the geographic area of Naples, Italy, the knowledge, beliefs, and coverage regarding the vaccines among a sample of parents and the associated factors.

The findings of this survey show that, generally, respondents were not very knowledgeable since only 16% identified all vaccinations recommended during adulthood and, in particular, only one third knew of the tetanus/diphtheria/pertussis vaccine. The inadequate knowledge can negatively affect the adults’ access to healthcare preventive services for the immunization leading to a poor control of the vaccine-preventable infectious diseases. However, participants have a positive attitude regarding the usefulness of the vaccinations and a large majority perceived the vaccine-preventable infectious diseases as being dangerous for them. Moreover, the positive attitude regarding the vaccinations confirmed previous investigations conducted by some of us in the same geographical area among different at-risk groups of the population [24,25,26].

It is important to underline that, despite this positive attitude, only a small proportion of parents has been immunized with the vaccines recommended. This is very disappointing since it is well established that during the natural age advancement the phenomenon of immunosenescence occurs, the ability of the immune system is reduced, and an adequate physiological response to infectious diseases and the immunity acquired in childhood is not guaranteed. Therefore, policy makers and healthcare professionals should make efforts to plan targeted information interventions and to guarantee an active and free vaccination program at all levels of care delivery. In this sample, the highest coverage was observed for tetanus (41.6%) and only 7.2% against measles. Conclusions of other similar studies regarding tetanus vary although the coverage was much lower than those observed in US (58%) [27], Canada (70%) [28], and Germany (78.2–73.1%) [2,29]. This indicates the importance of conducting such surveys in settings with different vaccination strategies. Reaching optimal vaccination coverage for measles is essential considering the recent outbreaks in Italy and in other European countries [30,31] due to suboptimal vaccination coverage among children, adults, and healthcare professionals. Despite very low vaccination rates, the majority of participants reported a positive attitude toward willingness to receive the recommended vaccinations. However, several reasons have been indicated for the unwillingness to receive the vaccinations such as the objection to the administration of the vaccines, perception of the vaccine-preventable infectious diseases as not being dangerous, and concerns about side effects of vaccines. Similar results were also seen in previous studies [32,33,34,35,36]. Therefore, this population should receive adequate information regarding the efficacy and safety of vaccinations and the dangers of several infectious diseases if contracted during adulthood. Moreover, information strategies are needed in order to increase the access of adults and the healthy population to vaccination services. 

A main objective of this survey was to provide an insight into the independent predictors of the outcomes of interest. Results of the multivariate analysis showed that respondents who had at least one chronic condition were more likely to know the vaccinations recommended in adulthood and therefore to be immunized. This may be due to the fact that such individuals are more likely to be informed because they have more frequent use of the healthcare services. This study indicated that the primary source for learning about vaccinations for the vast majority was physicians and this is in accordance with several previous studies, which showed that healthcare workers were the most trusted source to acquire health-related information [8,34,37,38,39]. Moreover, those who had used this source had a positive attitude toward willingness to receive vaccinations recommended although physicians’ advice had no effect on having received at least one vaccination recommended during adulthood. This apparent failure to provide the adequate information regarding vaccine delivery should be addressed since giving this information does in fact result in higher vaccination coverage. Several studies conducted in the same geographical area suggested that if physicians provide vaccination information, populations’ knowledge, awareness, and behavior within the population would also increase [26,34,37]. Therefore, given the observed low immunization coverage, it is essential that since physicians play a key role in providing individuals with access to evidence-based information and public health approaches for reducing the burden of infectious diseases they must take more time and attention to help adults make informed choices about vaccination. Indeed, physicians should increase their efforts to reduce parents’ vaccine hesitancy, concerns, and misconceptions, through adequate communication about vaccination and making easier the access to the healthcare preventive services. Finally, being a healthcare professional was a predictor of having a higher level of knowledge and of having received at least one vaccination recommended during adulthood, and this may be explained by the fact that healthcare workers have a greater likelihood of having experienced cases of vaccine-preventable infectious diseases. This was unsurprising given that this significant association has been observed in past studies [40,41].

This survey has some limitations that should be taken into account in order to make a correct interpretation of the findings. First, the cross-sectional methodology of this study limits the possibility to determine the directionality of the association or the causal relationship between the outcomes of interest and predictors. Second, all measures were made on the basis of participant self-reporting, with no attempt to independently verify respondents’ information; thus, as with all similar surveys, social desirability and recall and response bias are possible. To reduce the potential for social desirability bias in responding, the survey was anonymous with no identifying data collected. Third, the sample was represented by parents of school age children recruited in one geographical area, and therefore, the generalizability of the findings to the rest of Italy needs to be established.

## 5. Conclusions

The level of knowledge and coverage rates for the vaccinations recommended in the adult population are a critical issue, and therefore, these results underline the need for greater efforts by policymakers and healthcare providers to increase the parents’ knowledge, and it is crucial that healthcare providers have a high level of knowledge and favorable attitudes in order to increase vaccination coverage.

## Figures and Tables

**Table 1:** Main characteristics of the study population.

Characteristics	*n*	%
*Age, mean ± SD (range), years*	45.2 ± 5.8 (19–71)	
*Gender*		
Female	306	74.3
Male	106	25.7
*Number of children*		
1	65	16
>1	341	84
*Educational level*		
None or primary school	2	0.5
Middle school	57	14.1
High school	231	57.2
College degree or higher	114	28.2
*Marital status*		
Married	371	90
Other	41	10
*Employment status*		
Unemployed	129	34.6
Employed	243	65.4
*Working as healthcare professional*	24	5.8
*Having at least one chronic disease*		
Yes	26	6.3
No	385	93.7

Number for each item may not add up to total number of study population due to missing value.

**Table 2:** Participant’s knowledge about the recommended vaccinations.

Which Vaccination Is Recommended During Adulthood?	*N*	%
	Correct Response	
Influenza *^,^^	146	35.4
Tetanus/diphtheria/pertussis *	126	30.6
Measles/mumps/rubella *	110	26.7
Varicella *	77	19.7
Pneumococcal disease	56	13.6
Tuberculosis	54	13.2
Human papillomavirus	52	12.6
Meningococcal disease	42	10.2
Hepatitis A	36	8.7
Haemophilus influenza type b	29	7.1

* Recommended vaccination; ^ Recommended vaccination for patients with chronic conditions.

**Table 3:** Multivariate logistic and linear regression analysis results for estimates of associations of the different outcomes of interest among parents with several variables.

**Variable**	**OR**	**SE**	**95%CI**	***p* value**
Model 1. Knowledge of the vaccinations recommended to adults between 19–64 years of age				
Log likelihood = −131.67, χ^2^ = 71.81 (5df), *p* < 0.0001				
Having at least one chronic condition	28.8	16.18	9.62–86.63	<0.001
None or primary/middle/high schools	3.97	1.82	1.62–9.75	0.003
Working as healthcare professional	3.17	1.77	1.06–9.52	0.039
Having received information about vaccinations from physicians	2.68	1.33	1.01–7.09	0.046
**Variable**	**Coef.**	**SE**	***t***	***p* value**
Model 2. Having a positive attitude towards the usefulness of the administration of vaccinations recommended to adults between 19–64 years of age				
*F* = 4.42, *R*^2^ = 0.05, adjusted R^2^ = 0.04, *p* < 0.0001				
Lower number of children	–0.48	0.16	–3	0.003
Having received information about vaccinations from physicians	0.65	0.27	2.72	0.016
Females	0.55	0.27	2.03	0.043
Younger age	0.43	0.26	1.72	0.087
**Variable**	**OR**	**SE**	**95%CI**	***p* value**
Model 3. Positive attitude toward willingness to receive vaccinations recommended to adults between 19–64 years of age				
Log likelihood = −227.26, χ^2^ = 55.93 (5df), *p* < 0.0001				
Having received information about vaccinations from physicians	3.15	1.05	1.63–6.08	0.001
Who considered the administration of the vaccinations as being useful	1.31	0.05	1.05–1.61	0.013
Older age	1.42	0.45	0.76–2.64	0.075
College degree or higher	1.62	0.46	0.93–2.83	0.084
Who considered the vaccine-preventable infectious diseases as being dangerous	1.06	0.06	0.94–1.21	0.321
Who considered the administration of the vaccinations as being dangerous	0.95	0.05	0.86–1.05	0.336
**Variable**	**OR**	**SE**	**95%CI**	***p* value**
Model 4. Having received at least one vaccination recommended to adults between 19–64 years of age				
Log likelihood = −154.10, χ^2^ = 25.30 (5df), *p* < 0.0001				
Having at least one chronic condition	7.38	3.74	2.73–19.94	<0.001
Who considered the administration of the vaccinations as being useful	1.33	0.15	1.06–1.67	0.013
Working as healthcare professional	2.77	1.36	1.05–7.26	0.038
Who considered the administration of the vaccinations as being useful	1.1	0.06	0.99–1.24	0.07
College degree or higher	1.41	0.44	0.76–2.61	0.275
